# A Case of Eagle Syndrome in a Chiropractic Patient

**DOI:** 10.7759/cureus.38426

**Published:** 2023-05-02

**Authors:** Leonard F Vernon

**Affiliations:** 1 Clinical Sciences, Sherman College of Chiropractic, Spartanburg, USA

**Keywords:** non-specific neck pain, advanced imaging, trauma, eagle syndrome, chiropractic

## Abstract

Eagle syndrome is a rare condition that is characterized by, among other things, pain in the face and neck, with the majority of cases being unilateral and isolated to the lower jaw. It is not uncommon for the pain to radiate to the ear. Symptoms can be constant or intermittent and may increase with yawning or rotation of the head, causing Eagle syndrome to be frequently misdiagnosed. The objective of this report is to summarize the symptoms, diagnostic workup, necessary imaging, and management of Eagle syndrome.

## Introduction

The styloid process attaches the temporal bone of the skull and abuts to the styloid foramen, where it has numerous attachments, including the stylohyoid and stylomandibular ligaments and styloglossus and stylopharyngeus muscles. Derived from the Greek word “stylos”, which implies the pillar in Greek. The length of the styloid process has been reported by some authors to range between 15.2 mm and 47.7 mm [1,2], with various authors labeling an elongated styloid process as anything >30 mm while Wat W. Eagle, an otolaryngologist, whom the syndrome is named after, believed that a length >25 mm is considered elongated [3]. A radiographic study by Dayal et al. seems to confirm Eagle’s assertion [4]. Watt found the incidence of elongated styloid process is 4% in the general population, of which only 4%-10% are reported to be symptomatic, with a female-to-male ratio of 3:1. It is usually reported in adults after the third decade of life [5,6].

In 1652, Pietro Marchetti first described an elongated styloid process related to an ossifying process of the stylohyoid ligament [7]. In 1937, otolaryngologist Wat W. Eagle coined the term “stylalgia” to describe the pain associated with this abnormality [8]. Wat W. Eagle (1948) hypothesized that the formation of scar tissue around the styloid apex after tonsillectomy caused compression and straining of the neurovascular structures present in the retro styloid compartment affecting stretches of cranial nerves V, VII, IX, and X [9]. This has since been expanded to include even minor cervical spine trauma [10,11]. No matter the what is the etiology, patients almost uniformly report all or some of the following symptoms: foreign body sensation, pain referred to the ear, and dysphagia [12]. Saccommano et al. [13] found a correlation between Eagle syndrome and traumatic events and suggested two possibilities: a traumatic event could fracture the already elongated styloid process or calcified stylohyoid ligament; trauma itself triggers the pathophysiological mechanisms that lead to lengthening of styloid process or calcification of stylohyoid ligament and therefore the typical symptoms [14].

The same authors (Saccommano et al. [13] and Todo et al. [14]) found that the carotid artery type of Eagle's syndrome presents with other symptoms, such as migraines and neurological symptoms caused by irritation of the sympathetic nerve plexus. Eagle’s Syndrome has been shown to mimic osteoarthrosis of the temporomandibular joint; thus, the misdiagnosis of temporomandibular syndrome is frequent. The relationship of the styloid process to both the carotid artery and neurological structures in the region are setting for the perfect storm. If the internal carotid artery is compressed, then ipsilateral headaches can occur. If the external carotid artery is compressed, then there can be pain in the temporal and maxillary branch areas. A more significant danger with elongated styloid, although rare, is the possibility of carotid artery dissection, stroke, and sudden death due to this syndrome, as has been noted by multiple authors [15]. Sudden death is due to mechanical irritation of the carotid sinus by an elongated styloid process which may cause the heart to stop, resulting in cardiac arrest [16-18]. 

## Case presentation

A 61-year-old African American male with no previous history of neck or jaw pain male presented to a chiropractic office two months following a traumatic incident to the cervical spine. His past medical history was non-remarkable, and his only prior major surgical procedure was a total arthroplasty of the left shoulder.

On examination of the cervical spine, the range of motion was found to be limited and moderately painful, especially in bilateral rotation as well as on extension. Additional symptoms included left sided mandibular pain with occasional headache. The patient indicated that the headaches and jaw pain were new, and he denied any history of previous discomfort in these areas. He indicated that he was recently seen in a hospital emergency department (ED) following an unexplained episode of syncope. He received a CT examination of the head, which was reported as negative. He did remember striking his jaw when passing out; and based on the history and the examination, which did reveal tenderness on palpation of the temporomandibular joint (TMJ) as well as discomfort of the joint on opening of the mouth, a preliminary diagnosis of post traumatic temporomandibular syndrome as well as traumatic cervical spine injury was made. Eagle syndrome is often confused with other temporomandibular disorders (TMD) and, due to its symptoms, is often missed when diagnosing facial pain [19,20]. Temporomandibular syndrome has been shown to cause cervical spine pain, and both are frequently treated by chiropractors [21].

**Figure 1:**
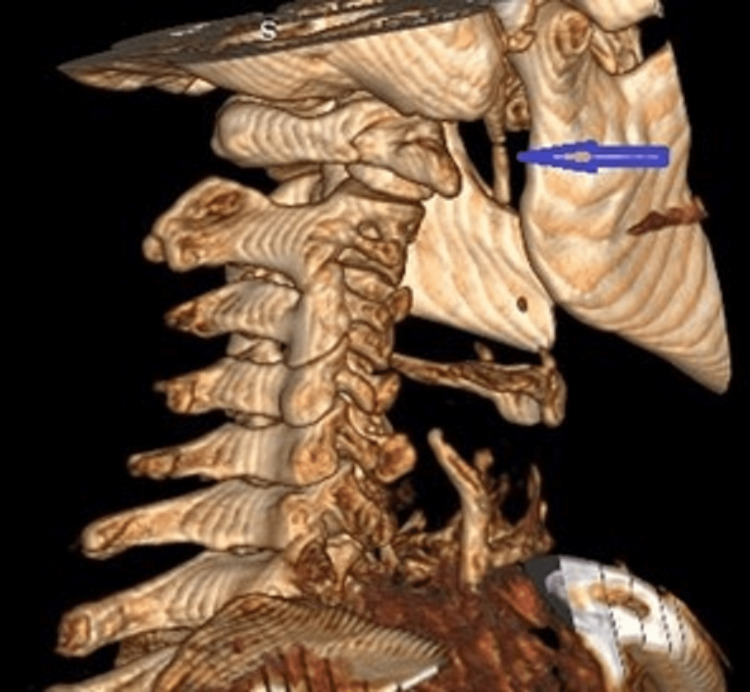
3D reconstruction of the cervical spine (lateral view) The image is showing elongated styloid process with pseudo articulation (blue arrow).

Following a period of two weeks of conservative care, including cervical spine manipulation, as well as intraoral massage and manipulation of the TMJ, which in retrospect had the potential of additional injury of the cervical spine, the patient had little relief and a CT scan with 3D reconstruction of the cervical spine was ordered (Figure 1).

**Figure 2:**
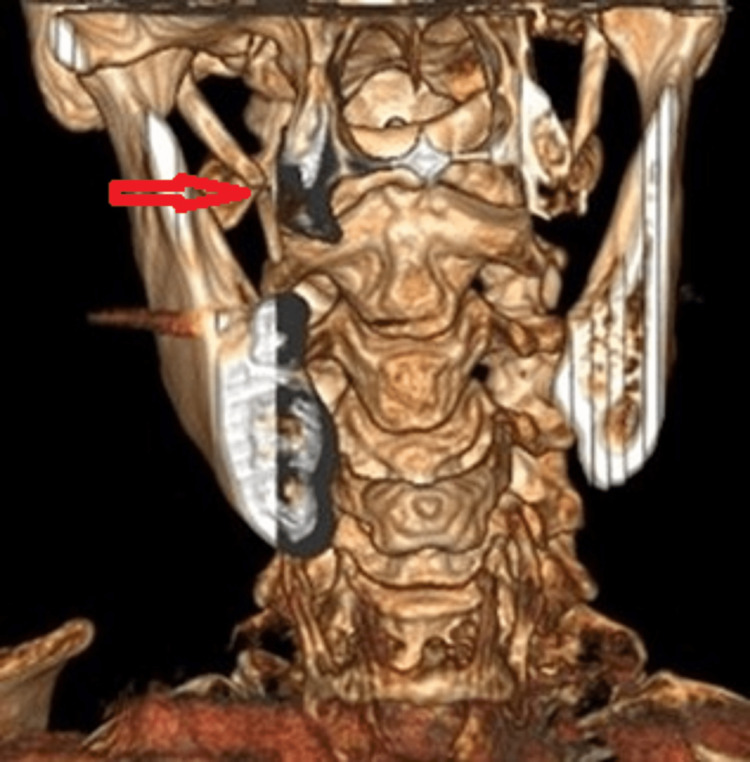
3D reconstruction of the cervical spine (AP view) The anteroposterior (AP) view image is showing an elongated styloid process on the right compared to the left (red arrow).

**Figure 3:**
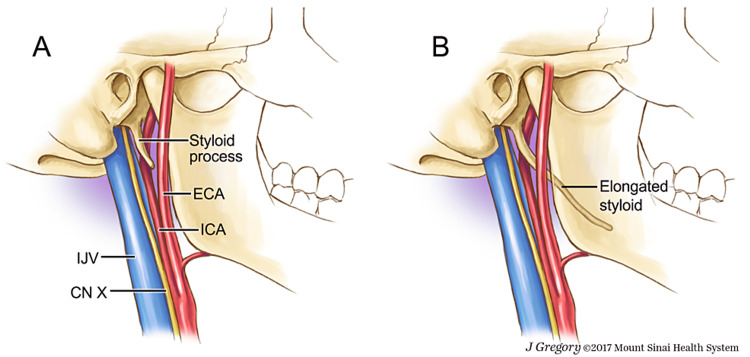
Anatomical relationship of styloid process to other structures in the region This illustration clearly outlines the importance of understanding the anatomical landmarks both with and without an elongated styloid process. One can see the proximity to neural and vascular structures in the region and the potential for symptoms with elongation. Image credit: This image is used with permission from ©Mount Sinai Health System.

The results of this examination revealed an elongated styloid process with a pseudo articulation of the right side (Figures 2, 3). The patient was referred for an ear, nose, and throat (ENT) consultation that resulted in a recommendation for surgery which the patient refused. Conservative treatments, including analgesics, antidepressant medications, anticonvulsants, transpharyngeal injection of steroids and lidocaine, diazepam, nonsteroidal anti-inflammatory drugs, and the application of topical heat have been recommended [22].

## Discussion

This case demonstrates the complexities of diagnosing cervical spine pain and craniofacial pain. While most cases that present to the practitioner’s office will be of a benign nature, such as cervical strain and sprain or a temporomandibular joint problem, there are certain red flags that should alert the practitioner to the possibility of a more serious condition. In this case, the history of a syncopal episode and the lack of response to conservative care led to the ordering of advanced imaging studies, including 3D reconstruction of a CT scan of the cervical spine, which revealed the pathology and aided in the differential diagnosis.

Chiropractors are portal-of-entry healthcare providers who are frequently sought out to treat neck pain. Studies have shown a growing trend of the US adults utilizing chiropractic care with neck pain accounting for nearly one third (30.2%) of all visits to a chiropractor [23]. Neck pain is also one of the leading causes of disability in the US [24]. This is an example of the multitude of pathologies that, although rare, can and will present to the chiropractor's office with a chief complaint of neck pain.

This case illustrates the importance of a detailed case history and physical examination, and while in this case both were done, a misdiagnosis still occurred and could have had serious consequences for both the patient and the doctor. Many practitioners are under the mistaken belief that since the patient was seen in an emergency room and discharged that the patient is, in essence, "cleared" and that only a perfunctory history and examination are required. In this case, because the patient reported an episode of syncope, a CT of the head was performed and reported as negative, a result that could lead the treating chiropractor to believe there were no contraindications to manipulation or other treatment modalities. This case points out the importance of performing your own thorough history and examination as well as the utilization of advanced imaging. In this case, CT scan of the cervical spine with 3D reconstruction was performed, which is considered the gold standard [25].

## Conclusions

The mere presence of an elongated styloid process does not automatically confirm a case of Eagle syndrome; however, as in the above case, the elongated styloid process that was accompanied by autonomic symptoms (syncope), as well as facial and cervical spine symptoms, confirmed the case. While the pathophysiological mechanism of symptoms is still debated, it is clear that the elongated styloid pro­cess can cause compression or irritation of one or more of the various anatomical structures in the region. This compression and or irritation can cause a variety of symptoms which will frequently cause a patient to seek out chiropractic care, and these symptoms can range from simple neck pain or cervicofacial pain to cerebral ischemia symptoms.
